# Achieving consensus advice for paediatricians and other health professionals: on prevention, recognition and management of conflict in paediatric practice

**DOI:** 10.1136/archdischild-2018-316485

**Published:** 2019-04-18

**Authors:** Mike Linney, Richard D W Hain, Dominic Wilkinson, Peter-Marc Fortune, Sarah Barclay, Vic Larcher, Jacqueline Fitzgerald, Emily Arkell

**Affiliations:** 1 Women and Childrens, Western Sussex Hospitals NHS Foundation Trust, Worthing, West Sussex, UK; 2 Royal College of Paediatrics and Child Health, London, UK; 3 All-Wales Paediatric Palliative Care Network, Noah’s Ark Children’s Hospital for Wales, Cardiff, UK; 4 Oxford Uehiro Centre for Practical Ethics, University of Oxford, Oxford, UK; 5 Newborn care unit, John Radcliffe Hospital, Oxford, UK, Oxford, UK; 6 Paediatric Intensive Care Unit, Royal Manchester Children’s Hospital, Manchester, Manchester, UK; 7 Medical Mediation Foundation, London, UK; 8 Honorary Consultant in Bioethics, Great Ormond Street Hospital For Children NHS Trust, London, UK

**Keywords:** palliative care, intensive care, ethics, multidisciplinary team-care

## Introduction

Conflict can arise between health professionals and the parents of children not only where there is disagreement on the withdrawal or withholding of life sustaining treatment, as seen in recent high-profile cases^1 2^ but also in more general routine care. In this paper we attempt to suggest practices which may reduce disharmony. Although, many of you will already incorporate such practices in your daily working lives, we felt it useful to collate this guidance covering prevention, recognition and management of situations when conflict clearly exists. It will not resolve all conflicts and clearly there will be other practices which are helpful but are not mentioned here, similarly there are further reflections needed on recent cases^3^ There is limited research in this field so we have taken evidence from clinicians, parents, parent advocates, ethicists and mediators of conflict in writing this document. As healthcare professionals involved in the care of children and young people, every decision we take will always have the best interests of the child at heart. Decisions on care, including the withdrawal of treatment, as far as is possible, should always be made with the involvement of parents. In the majority of cases in these decisions are made jointly by parents and clinicians.^4^

However, disagreements leading to conflict can sometimes develop between professionals and families in the context of critical illness both in children and adults.^5 6^ End-of-life decision making and communication failure are the common areas of dispute.^6 7^ Conflict is also prevalent in children’s inpatient wards^8^ where staff report communication breakdown, disagreements over treatment and unrealistic expectations as the most common causes. A contributor to this may be the fact that the parents and carers of children in hospital report receiving conflicting information from health professionals.^9^ With this in mind, the guidance, although primarily developed with the withdrawal of life-sustaining treatment, will have relevance in many other scenarios.

**Key points**

- Avoid giving inappropriate expectations.
- Use palliative care teams early, not just for end of life care, but when treatment options are being discussed.
- Recognise that parents will be under severe stress and offer psychosocial support especially those with children with complex needs or conditions which are life changing or life limiting.
- Equally support practitioners by the bedside who may be caught up in the conflict.
- Assign Lead Clinician role to ensure continuity of information and understanding.
- Develop skills within your service to recognise the development of conflict.
- External expert advice may be helpful, including ethical and legal services and consideration of early involvement of mediation services.

The increasing prevalence of both complex and life-limiting conditions in individuals aged 0–19 years,^10^ the increasing availability of advanced forms of life-sustaining treatment and the large amount of information online about innovative but unproven treatments for serious illnesses^11^ are factors that may produce increased risks of conflict. Also, disagreements may arise in relation to the clinical facts, for example, what treatment is technically feasible, against sincerely and firmly held beliefs and values as to what treatment *should* be given.

The Royal College of Paediatrics and Child Health (RCPCH) has previously produced guidelines on limitations of treatment, including guidance around decision making and managing disagreement.^12^ However, recent cases of entrenched disagreement have been associated with intense media coverage and demonstrations outside children’s hospitals.^13^ This can have profound effects on staff, children and their families.

All healthcare professionals working with children and young people encounter parental concerns about treatment plans from time to time. Experience teaches us that disagreements in healthcare can usually be managed by a process of shared decision making with active early involvement of parents and whenever possible with the child/young person. The importance of honest and open communication with families as early as possible cannot be understated. In most cases, even in areas of persistent disagreement, resolution can be achieved by careful listening and understanding and where possible early involvement of mediation services. However, in some circumstances, discussion between healthcare professionals and the family is not successful, and there is a need to take both legal and perhaps ethical advice in order to act in the best interest of the child. Recourse to the courts is a final step, but this whole process is time consuming and protracted with a profound psychological impact on families and staff. Avoidance of conflict is not always possible, but we would wish to strive to achieve this by careful planning and management. We believe palliative care teams have a lot to offer in these discussions and their early involvement is beneficial.

In this document we have tried to summarise practical recommendations for those working with children and young people both to prevent and manage conflict, in effect to achieve consensus in decision making with families. Since evidence about the best ways of achieving consensus in healthcare is limited, these recommendations, endorsed by the RCPCH, are necessarily based on opinion and experience. They are neither exhaustive nor obligatory, as every situation will have its own unique context and solution. Where relevant, we have extracted narrative from the RCPCH 2015 framework document.^12^

In all situations, the ethical and legal basis for all decisions is that it is the best interest of the child that is paramount. Sometimes parents and health professionals disagree about what those interests may, be but it is important that parental wishes and views are listened to and respected in discussions with the clinical team.

Clinicians should be aware that courts cannot dictate treatment to be given to a child rather that the ‘choice of treatment is in some measure a joint decision of the doctors and court or parents’ as explained by Lord Donaldson.^14 15^ It is likely that where decisions are deemed by courts too difficult for either judges or clinicians to resolve, the choice should be one for those with parental responsibility, as per Lord Justice Waite.^16^ It is also recognised^17^ that the court’s clear respect for the sanctity of human life must impose a strong obligation in favour of taking all steps capable of preserving life, save in exceptional circumstances. The courts will balance for the best interests of the child, the principle of the sanctity of human life against any question of the discontinuation of life sustaining treatment.

A new conflict management framework to help staff for recognise and de-escalate conflicts in paediatric healthcare has been developed^18^ and is currently being tested in four UK hospital sites during the autumn of 2018 and early 2019. Results from an initial pilot carried out in one oncology ward in a children’s hospital in Perth, Western Australia, in 2017 was positive.^18^ Further data on use of the framework are now being collected at the four UK pilot sites to see if it can be helpful in the UK healthcare setting.

## Guidance

The following sections set out guidance and some practical suggestions to support paediatric healthcare professionals to identify, prevent and manage potential conflict situations at the earliest opportunity.

## Preventative management

Try to avoid giving families unrealistic expectations of clinical outcome. Managing expectations while still escalating care is important.

Early involvement of the local palliative care services is linked to better outcomes.^19^

Palliative care services may be best at supporting the decision-making process. Promoting the understanding that their role is not just ‘end of life’ care but part of the active support process for children and young people with life-limiting conditions to ensure their quality of life. This includes managing transition of care as the clinical situation changes.^20 21^ Close communication between teams is essential at all times.

The introduction of palliative care should not be left until a decision is made to withdraw or withhold life-sustaining treatment. A palliative care team can support the child and family to live in the knowledge of an uncertain future. They can provide practical and emotional support for day-to-day care and support for symptom management. (RCPCH 2015, section 3.2.6, ref 12)

Offer early psychosocial support to children and families, especially those with children with complex needs or conditions that are life changing or life limiting.

Make sure time is set aside to listen to parents and to understand their perspective, especially where disagreements arise. Provide them with consistent and timely feedback on their areas of concern. Ensure that different clinical staff are not giving conflicting information to parents and importantly that bedside nurses and therapists are kept informed of and understand the rationale for any changes to treatment plans as they are the ones who spend most time with parents.

Remember that all healthcare professionals involved in these complex situations particularly those by the bedside may themselves need extra support, the ability to access this support when required is essential.

Be sensitive to the fact that parents may act in difficult or unusual ways because of the stresses they are encountering, and this may relate to their own previous experiences. They may become desperate, anxious and angry if they believe that health professionals are failing their child, perhaps by not considering unproven treatments no matter how apparently futile. This leads to frustration as their decisions and choices may appear not to be heard.

Try to ensure that discussions with parents are holistic and that opinions offered by health professionals are made within that context. This requires all aspects of the child’s current and past experience to be taken into account, including parental and wider clinical views about the child’s current quality of life. At the same time, although the child remains the primary focus, recognising the quality as well as the prolongation of life, the family’s understanding, wishes and needs should be included. Ensure equally that the family understands that the health professionals over-riding responsibility is to the welfare of the child and that is the basis of decision making. Excellent literature is available to help families understand and make these critical care choices.^22 23^

Ensure the full multidisciplinary team is aware of all important aspects of a child’s care, particularly specialists who are not routinely part of the team and who may have a misunderstanding of expectations. Consider whether colleagues from referring hospitals also need to be involved.

Information offered to families may change as the clinical team changes on a weekly basis, for example, due to the consultant of the week rotas. In complex situations, particularly where admissions have been prolonged, it is appropriate to assign a lead clinician role for the child. The lead needs to be compassionate and caring as well as having the appropriate level of knowledge and understanding of the child’s condition. The ‘Lead’ role for children with complex or life-changing conditions can:

- Act as liaison between family and medical/nursing team supporting access to the full multidisciplinary team.
- Recognise and acknowledge a family’s understanding and expectations and if misunderstandings develop be prepared to intervene to resolve any uncertainties.
- Be responsible for the overall care of the child.
- Help coordinate a consistent clear message to the family by all health professionals avoiding potential areas of confusion

## Identification of conflict

Teams need to have a robust strategy to spot early signs of conflict developing between families and health professionals. Development of a breakdown in relationships can be seen by:

*Avoidance behaviour*: parents avoiding health or specific health professionals, health professionals themselves avoiding the family.

*Demanding or controlling behaviour*: parents allowing only specific professionals to look after their child, questioning expertise or feeling the need to record all conversations. Health professionals finding every conversation with families a battle.

*Micromanagement*: parents requesting authority and review on every aspect of care.

As breakdown escalates, this can lead to:

*Entrenched positions*: development of separate camps, an ‘us and them’ attitude.

Eventually, the conflict itself becomes the focus.^8^

Forbat *et al* 8 identified three levels of conflict:

- Mild with poor management of relationships with the family,

- Moderate where a deterioration of trust occurs leading to

- Severe with disintegration of working relationships

## Early management for when conflicts start to arise

Ensure advice and guidance is available to families where disagreement arises. This could be through Patient Advice and Liaison Service (PALS) but also through internal resources within units. It is important that units develop their own specific support and guidance capabilities:

In a relatively small number of cases, disagreement over treatment decisions may lead to escalating conflict. In such cases, external advice and/or conciliation from one of a number of sources may prove helpful to the parties. The engagement of a number of supportive groups, such as Clinical Ethics Committees (CECs), Hospital Chaplaincy and PALS, has helped avert many potential court hearings. (RCPCH 2015 section 3.3.6, ref 12)

Guidance could include:

- Offering families’ religious support, PALS and palliative care

- Advice on how to access senior members of the service if not already arranged.

- Information on how decisions are made and in particular how ethical decisions are made and why requests for support in decision making are made to clinical ethicists.

- Offering families’ access to available mediation services.

- Support and understanding of the court process if that is necessary

Although ethical decision making is a continuous process for clinicians, specific requests for ethical advice may be sought outside of the clinical team when difficult ethical decisions need to be resolved. Some hospitals will have their own ethical service to use; for others, it is important to determine where, when and how that advice could be gained. By their nature, these processes may take time so early referrals when needed are encouraged.

Where disagreement remains, clinicians need to know how, when and where to access expert legal advice, for example, in managing conflict around withdrawal of life-sustaining treatment. This may mean going beyond the hospital’s usual legal services.

In most cases, the healthcare team and parents will come to agree over a course of action. If agreement cannot be reached, legal advice should be sought from specialist healthcare lawyers. (RCPCH 2015 section 3.3.6 E, ref 12)

When under stress, everyone involved may act in a different, difficult or indeed challenging way. It is important to meet with parents early to both recognise their problems and acknowledge the difficulties that maybe arising because of any disagreements. Such a discussion should be led jointly by the senior lead clinician and nurse; it may be helpful to have independent family support such as PALS present.

Remember to ensure all of the multidisciplinary staff are informed. This will include early and comprehensive multidisciplinary team meetings.

## Escalation

The following steps may be needed:

In extreme circumstances where it is believed parental behaviour is having an effect on clinical care, this will need to be managed. Examples of management in these scenarios could include agreed behavioural and communication contracts between parents and the clinical team. Support will be required to the clinicians and nursing teams involved.

Seeking second or further opinions: parents may request this where there is disagreement. It may also be considered early by the clinical team if treatment options are unclear and can be a measure of holistic understanding by the clinical team. This could be offered from internal or external colleagues. Those providing second opinions should have the necessary skills and expertise to do so. They should be given all the materials and support they require to fulfil their role.

Seeking a second opinion is not a legal requirement. It does conform to principles of good ethical decision making and the due process that good clinical governance requires. (RCPCH 2015, section 3.3.2, ref 12)

Requests for moving a child to different hospitals are made by some families in extreme situations for second opinions. The reasons behind these requests should be fully explored by listening carefully to the families and attempts made to reconcile differences of opinions. Using mediation services may be of help at this stage if not already accessed. Meeting representatives from a potential receiving hospital if that is possible may help build understanding between the local team and families. If the request for transfer remains and is appropriate due to the seriousness of the condition, then the next actions should be carefully considered, understanding the best interest of the child should be realised but listening to parental concerns.

If resolution is not possible and it may not be, parental requests for opinions and/or ongoing care may be requested from overseas jurisdictions for children with the most severe life-limiting conditions. Advice from senior professionals within the field and ethicists should be sought and shared with the family. If the assessment remains that it is not in the child’s best interest to be transferred, then further legal guidance may need to be sought.. The legal system will remain the final decision maker in these circumstances.

Formal support services for staff during and postconflict situations needs to be available.

## Conflict management

During episodes of conflict the following actions may need to be considered:

Ensure all media requests are managed by the designated and appropriately trained staff. Experience demonstrates that resorting to ‘no comment’ is unhelpful and the media should be engaged positively without breaking confidentiality of events not in the public domain. Hospitals may need to seek external advice on handling the media in these difficult circumstances.

Provide advice to all staff on the importance of avoiding engagement on social media except by the designated communication team from the hospital. Advice on social media for health professionals is available from the General Medical Council and the British Medical Association.^24 25^

Limit unsolicited second opinions by recognising what facts can be released by the hospital.

Recognise that social media may create popular interest and could lead to demonstrations impacting on daily clinical care and therefore consider how this could be best managed.

Support families in understanding the possible impact and effects on their personal lives if press or social media involvement is invited.

Consider how disruptive behaviour in clinical areas may be managed. This may mean family contracts. If clinical areas are being compromised, then exclusions may be necessary as a final resort although a dialogue with families must be maintained at all times.

Hospitals need to be able to actively support and advise clinicians if group referrals are made to the GMC.

## Services requested to offer second opinions

If units are asked to support families from other units, they should be able to:

Offer advice and support to paediatricians if they are requested to give a second opinion.

Second opinions can be most successful when families have a chance first to meet the clinician/s giving the second opinion before they see the child. Opinions given by telephone may be appropriate in some situations but not all and may not in themselves resolve any conflict.

Offer guidance on receiving children when conflict has arisen and when transfer is deemed suitable by all, taking into consideration the consequences of this for the child.

Recognise conflict is difficult to manage and needs discussion with the whole team and an action plan that is designed to protect the interests of the child while listening to parental concerns and hopefully reduce the risk of conflict escalation.

## Conclusion

Conflict is damaging, stressful and emotionally challenging for all involved. Taking the correct early steps may prevent early disagreements reaching conflict. If conflict is reached, families must continue to be supported even if there is a breakdown of trust between families and clinicians. As the voice of the child and what is in their best interest remains paramount, the families’ wishes and needs must also be taken into consideration.
